# Climate and Human Health: The Impact of Climate Change on Vector-Borne Diseases, Paphos, Cyprus (17–19 October 2012)

**DOI:** 10.1179/2047772413Z.000000000161

**Published:** 2013-12

**Authors:** Joanna Waldock, Paul E Parham, Jos Lelieveld, George K Christophides

**Affiliations:** 1 The Cyprus Institute, Cyprus; 2 Imperial College London, UK; 3 Max Planck Institute for Chemistry, Germany

## Introduction

Climate change is expected to impact widely upon human health, including changes in the geographic distribution of vectors that carry severe diseases such as malaria, Dengue fever, Chikungunya, and others. Countries in the Eastern Mediterranean and Middle East (EMME) have historically been devastated by such diseases, and may represent regions at particular future risk given projections of greater changes in climate than average global estimates. They also border regions where vector-borne diseases (VBDs) are endemic. The Cyprus Institute and the Grantham Institute for Climate Change at Imperial College London recently established a close collaboration and modelling consortium to study the impacts of climate change on VBDs in the EMME, and facilitating this workshop to establish key challenges in the field represented an important first output of this collaboration. Over three days from 17th to 19th October 2012, researchers from Cyprus, the UK, several European countries, the USA, and Israel provided an overview of recent research into climate impacts on VBDs, discussing, in particular, how better projections of climate change impacts on these diseases may be obtained in future work. The development of tools such as mathematical models to help plan strategies for the control of both vectors and the diseases they transmit formed a key focus; the greatest threats to the EMME were also identified and the climatic sensitivity of these diseases was discussed at length.

## The changing climate

Session 1 focused on the changing climate of the EMME established through progress made in developing improved regional climate models (RCMs) to investigate future changes in meteorological conditions.

Jos Lelieveld (The Cyprus Institute, Cyprus) gave an overview of the projections from a downscaled RCM, demonstrating a statistically significant increase in summer temperatures across the region that are projected to be greater than global averages. Warming trends across the region are very strong, with the warmest current conditions representing the coolest future conditions (without even overlapping in some cases) by the end of the 21st century. Winter rainfall is expected to decrease in the majority of the region (although this drying trend is not statistically significant), raising concern over future water usage for some countries in the Middle East (ME). Some increases in rainfall are projected in the southern ME, as the tropical belt may extend northwards, although it should be noted that observational precipitation data is particularly poor for the EMME, which, in turn, makes it difficult to test climate models with accuracy. Overall, the low reliability of drying trend projections may occur due to the poor quality of precipitation data and the discrepancy between observed data and model output resolution.

**Figure 1:**
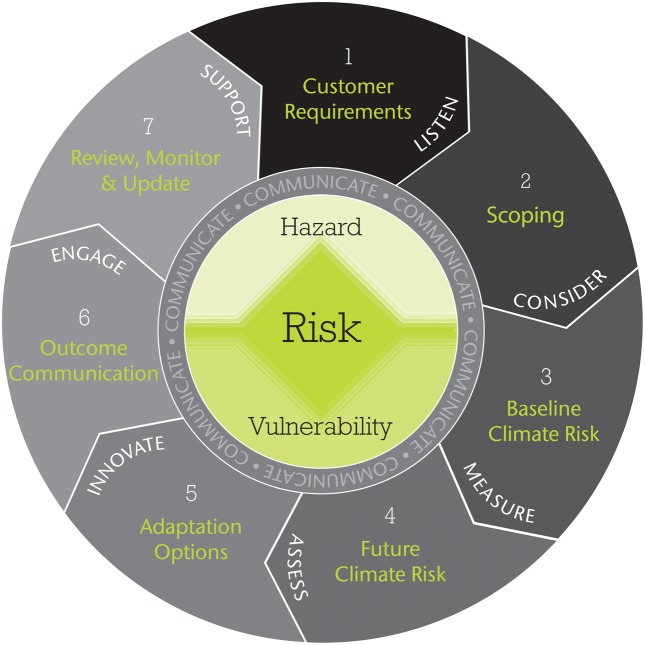
The Climate Impacts and Risk assessment Framework (CIRF) developed by the UK Met Office (reproduced courtesy of the UK Met Office)

Debbie Hemming (The Met Office, United Kingdom) discussed methods for improving the quantification of climate change using a risk-based approach (Fig. 1). Key in quantifying the uncertainty in modelling studies is the use of multi-model ensembles (MMEs). MMEs allow differences between distinct climate models to be visualised, for example by using colour strength as a reflection of agreement between models. MMEs demonstrate strong agreement in future drying in Greece, Cyprus, and Turkey, as well as projected rainfall increases in the southern ME. Regions where model agreement is weak may reflect poor observation data on meteorological conditions; Egypt is a particularly notable example.

Overall, this session highlighted the projected warming and drying of the EMME, the power of MMEs for understanding and quantifying uncertainty within climate modelling, and the need for better quality meteorological observation data for the evaluation of models (with particular reference to precipitation). It was unanimously agreed that precipitation is the most poorly-captured factor in climate modelling. An important topic of discussion was also the scale of climate models used in studies of biological systems. There is a large gap between the scale of typical global climate models (GCMs) and the microclimatic scales that often affect the dynamics of biological systems (such as the local habitats of mosquitoes). The need to initially move from global scales to higher resolution RCMs was therefore highlighted, along with the desire to improve model accuracy over complex topographical regions, but there still remain significant gaps between RCMs and the biological scale of species habitats. It was recommended that the requirements from biological perspectives first need to be defined, followed by an assessment of the need for ground measurements. A combination of ground measurements and model predictions may help to better capture scales important to biological systems than modelling alone.

## Insect vectors of disease and climate change

Session 2 focused on the impacts of climate change on vectors of human disease in the European Union (EU) and EMME. Francis Schaffner (University of Zurich, Switzerland) gave an overview of the current distribution of insect vectors within the EU. Insect vectors in the EU include multiple mosquito species (vectors of numerous viral diseases and malaria), sandflies (vectors of leishmaniasis), and ticks (vectors of numerous bacterial infections and viral diseases).

Of particular interest are a number of invasive mosquito species that are vectors, or potential vectors, of VBDs. Perhaps the most widely known is *Aedes albopictus*, the Asian Tiger mosquito, which invaded Europe 30 years ago and has become established in Italy, Croatia, southern France, and parts of Spain; recent expansions also include establishment in Greece and Southern Russia. *Aedes albopictus* was responsible for a small local outbreak of Chikungunya virus (CHIKV) in Italy in 2007, and concerns remain for local transmission from imported cases. *Aedes aegypti*, historically present in Europe (but eradicated during the 20th century), has recently been reported in Southern Russia, and this species may also spread into Turkey and the Ukraine. *Aedes aegypti* is the primary vector of Dengue virus (DENV), yellow fever virus (YFV), and CHIKV, plus a potential vector of several other viral infections. Three further invasive *Aedes* species, *Ae*. *japonicas*, *Ae*. *koreicus*, and *Ae*. *atropalpus*, also recently invaded central and northern Europe and these species have been shown in the laboratory to be competent vectors of viral disease, although their role as vectors in the field is less-established. The overall picture suggests *Ae*. *albopictus* spreading in Southern Europe, *Ae*. *japonicas* spreading in Central Europe, *Ae*. *koreicus*, and *Ae*. *atropalpus* attempting to invade in different locations, and *Ae*. *aegypti* close to invading Europe via Russia.

Surveillance is vital in identifying areas at risk of VBD outbreaks, yet key gaps in vector surveillance remain, with countries such as Turkey providing little information. Mapping of disease vectors is, however, strong for certain countries, particularly for *Ae*. *albopictus*, but poor generally for tick species. Countries for which extensive vector data is currently lacking include Turkey, Romania, Ukraine, Austria, and large parts of Spain, Germany, Poland, and Hungary.

Jolyon Medlock (Public Health England, United Kingdom) spoke about the role of surveillance in VBD mitigation in the UK, noting that in the context of VBDs, clinical disease in humans is the tip of the iceberg of a complex multifactorial disease transmission cycle. Key challenges for preparedness and response to VBD outbreaks include (a) understanding the risk, which is currently limited by a lack of knowledge on key vectors, (b) assessing the risk, and (c) mitigating the risk, which requires evidence-based field research into environmental management that reduces either vector numbers or minimises contact between vectors and hosts.

Essential for all risk assessments of VBDs (and planning effective surveillance or mitigation programmes) is the need to understand the entomological and ecological aspects contributing to transmission, including vectors, bridge vectors, primary/secondary/amplification hosts, and the impacts of environmental factors on each of these. Two key points should be considered with respect to surveillance planning and undertaking risk assessments of climate effects on VBDs: the first is the importance of specificity; the factors affecting VBD are specific to individual diseases and relate to the ecology of their vectors and hosts. For example, wind patterns have little effect on the distribution of mosquito species (which tend to have short flight ranges and remain close to the ground), while midge and blackfly populations migrate hundreds of kilometres, so wind patterns are critical for movement of these vector species. The second point concerns the current lack of empirical data essential for understanding the specific impacts of climate on VBDs. Much of the entomological work in the literature is dated. When considering new strains of emerging VBDs and shifts in vector and disease distribution, out-dated empirical studies do not account for adaptation of vectors and pathogens. Lack of funding for new empirical studies is a major barrier.

An example of the specificity required for modelling vector populations was given by Joanna Waldock (The Cyprus Institute, Cyprus), who considered the impact of climatic factors on *Ae*. *albopictus*. A literature search was undertaken and data from empirical studies over the last 40 years were combined to elucidate the relationship between temperature, rainfall, and relative humidity (RH) on *Ae*. *albopictus* development and survival. The majority of studies related to temperature impacts on development and survival, and a consistent relationship between increased temperatures and faster development was found across studies. Higher temperatures also correlated with reduced lifespan in adults and temperature extremes were associated with increased morality in all life stages. Little data was available on the effects of precipitation and RH on *Ae*. *albopictus*. It should also be noted that temperature and RH are not independent variables, with low humidity leading to decreased survival, an effect which is exacerbated at higher temperatures. A systematic review of the available literature is ultimately required for (a) parameterising models of VBD transmission, and (b) identifying gaps in empirical studies that require new experimental data for parameterising vector models. Without the inclusion of species-specific parameters, vector models may lack accuracy and reliability.

## Climate impacts on VBDS

The third session focused on the impact of climate on VBDs (including viral and parasitic diseases). A number of different approaches may be used for investigating the link between climate and VBDs and several examples for different diseases were presented.

The pace was set by George K. Christophides who presented his thoughts about new risks of VBDs in Europe and the Mediterranean, which may originate from changing climatic and environmental factors but also from societal factors that in conjunction with the above can play elemental roles. He presented the history of the Chikungunya outbreak in 2005–2006, the largest ever arboviral outbreak recorded, which was soon followed by the alerting outbreak in Italy. This outbreak was caused by *Ae. albopictus* that besides Chikungunya can effectively transmit Dengue fever, yellow fever and various encephalitides. He emphasised that this mosquito is now established in the Mediterranean, and countries of the proximal Middle East, effectively occupying the empty niche created after the elimination of its sister container-breeder *Ae. aegypti*, and stressed that this region has been devastated in the past by VBDs, including malaria and Dengue fever. He specifically referred to *Ae. aegypti*, which caused one of the worst Dengue outbreaks in Europe in 1927–1928, where 90% case prevalence was reported in Athens, Piraeus, and other major cities in Greece. This outbreak was largely attributable to financial depression, lack of vector control measures, and high human concentration in squatter settlements and refugee camps following a Greco-Turkish war and population exchange. He ended this historic perspective by saying that the current socioeconomic situation may seem profoundly different from that described, however, the factors that caused that outbreak are comparable for many areas in the region.

Jane Messina (University of Oxford, United Kingdom) spoke about estimating the global burden of Dengue fever and projecting the future global burden using ecological niche modelling. One of the major issues in producing estimates of disease occurrence, particularly in Third World countries, is a lack of incidence data. Dengue remains a largely unreported disease and this study used a combination of incidence data, systematic literature reviews, and questionnaire responses from the INDEPTH network to assess countries for the presence of Dengue using a scale reflecting inherent uncertainty. Environmental data sets were used for Boosted Regression Tree (BRT) ecological niche modelling. BRT modelling allows for interactions between variables (here, climate data from WorldClim, vegetation index, GRUMP 2010 population data, and economic data) to generate the probability of (Dengue) occurrence on a global scale, and work in this area centres on using future climate scenarios to project how the probability of Dengue occurrence may change over time.

Shlomit Paz (University of Haifa, Israel) presented work on a regression analysis of West Nile Virus (WNV) and climatic factors during recent outbreaks of WNV in Eastern Europe and Israel. After a recent upsurge in WNV cases in Europe in 2010, this study investigated the link between several climatic variables (daily mean, minimum, and maximum temperatures, dew point temperature, RH, and monthly precipitation). A reference period of 1981–2010 was selected and compared to data from March to October 2010. Correlations between meteorological conditions and the onset of disease were investigated, incorporating the possibility of a lag between the build-up of vector populations and increases in disease transmission. The study showed that temperature was the strongest factor associated with increases in WNF, with high temperature anomalies associated with disease onset. Interestingly, this association also showed no lag between high temperature anomalies and disease onset in the southern, warmer countries such as Israel, but lag times increased moving northwards (1 week lag in Greece and up to 4 weeks lag in Russia). RH demonstrated weak correlation with disease onset and this can only be considered a weak indicator of outbreak risk in Europe. Precipitation showed inconsistent patterns and should also be considered a weak indicator of WNV onset in Europe. These results are consistent with reports that higher temperatures are a critical factor in WNV transmission, as well as demonstrating how meteorological anomalies can be used for possible early warning of disease onset.

Onchocerciasis, a parasitic disease transmitted by blackflies, was the subject of a talk by Bob Cheke (University of Greenwich, United Kingdom). Blackflies breed in fast-flowing rivers and possess considerably different ecology to mosquito species. Meteorological factors that impact on blackfly populations include rainfall (the high river discharge rates required for immature development are driven by rainfall), temperature (blackfly development is heavily-dependent on temperature), RH (although the thresholds are unknown), and wind patterns (which affect adult dispersal and migration patterns). Using empirical studies from Ghana, the development rate of immature blackflies (as a function of temperature) was calculated. This was used in a basic model to investigate how changes in temperature will affect the population dynamics of blackflies (using an empirical relationship between ambient and water temperature, so that a 3°C increase in ambient temperature equates to a 1°C rise in water temperature). Increased temperatures are predicted to increase the number of biting blackflies. In combination with deforestation (especially close to rivers), this could lead to changes in species composition, biased towards longer lived, more efficient vector species, along with an increase in biting flies. Conversely, decreases in rainfall and drought could have a negative impact on fly populations.

A key problem that is becoming increasingly recognised in the context of modelling VBD response to climate change is uncertainty, two sources of which arise from either (a) the structure of a model or (b) variation in empirical data used to parameterise a model. Addressing the issue of uncertainty within model projections, and investigating how this will affect users of model outputs, is becoming increasingly important. Andy Morse (University of Liverpool, United Kingdom) spoke about modelling malaria transmission under current and future climate conditions and the inherent uncertainty within a complex multi-factorial transmission cycles (such as that of malaria). This initially focused on results using seasonal forecast modelling, demonstrating an increase in seasonal forecasting accuracy for West Africa over the last 10 years, and providing an example of using seasonal forecasts with a climate-driven malaria model to predict disease outbreaks in Botswana. An important question remains as to how accurate model projections must be to be of use in making intervention decisions using early warning systems.

One way to address the uncertainty within climate-driven disease models projections is to use multiple disease models in combination with multiple climate models. Morse gave an example where five distinct malaria models were used in combination with five GCMs to project current and future malaria distribution across Africa. This type of analysis can be used to try and better understand the bias in distinct disease and/or climate models, and can give a degree of certainty within model outputs (for example, using colour to demonstrate regions where multiple models agree or disagree). The analysis presented suggested that the distribution of malaria is not expected to change dramatically under climate change (comparing 1980–2010 with 2069–2099), although model projections disagree regarding small expansions into South Africa and a slight northward expansion into Sub-Saharan Africa. The most consistent observation across model combinations is the expansion of malaria distribution into the highlands of Ethiopia. By combining multiple models in this way, degrees of uncertainty are more visible and can be helpful for guiding the users of model outputs.

**Figure 2:**
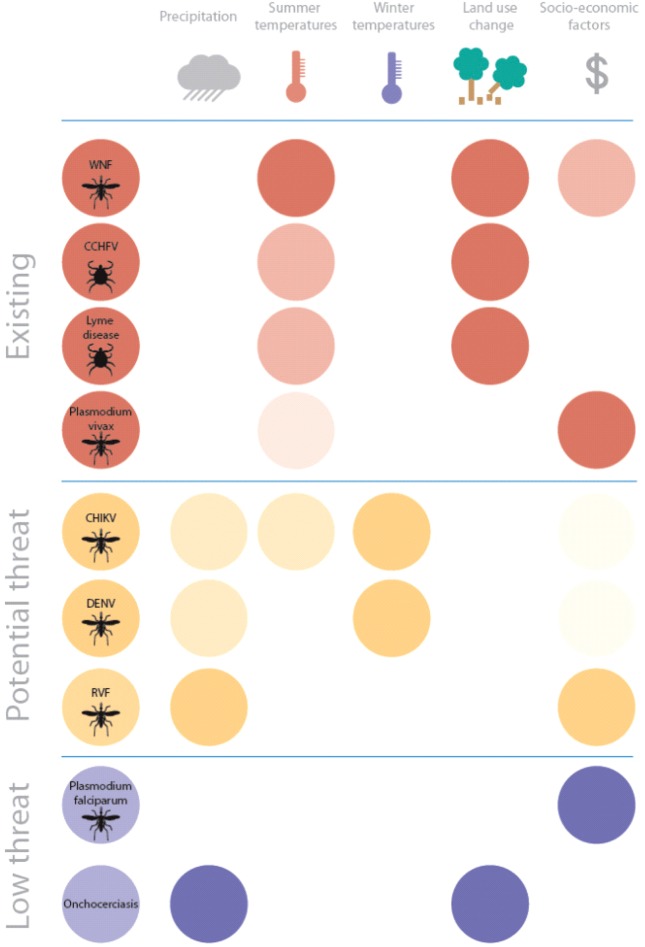
Risk factors affecting VBD transmission risk in the EU and EMME

## Climate-driven models of VBDS

The final session discussed advances in climate-driven models of VBDs, with a particular focus on malaria, the helminths, and Chikungunya.

Considerable challenges remain for modelling complex spatiotemporal VBD transmission. Paul Parham (Imperial College London, United Kingdom) spoke about model frameworks for VBDs, with a focus on the need for MMEs to (a) strengthen the robustness of model conclusions, and (b) identify and quantify uncertainties in model outputs. It was argued that structural differences between models are a strength, rather than weakness, of the modelling process and that ensemble approaches (ubiquitous in climate modelling) should also be applied to disease modelling, and a framework for combining the two was proposed. Developing reliable climate-driven vector population models is essential for, in turn, developing reliable VBD transmission models to inform future disease distribution (and intensity) under climate change. The example of a simple stage-structured model was presented, with mosquito species-specific parameterisation enabling wide application, and this was illustrated using previously-published work on developing a climate-driven *Anopheles gambiae* s.s. model and unpublished work on modelling the population dynamics of *Ae*. *albopictus* and *Culex quinquefasciatus*. These vector population models can be locally tested to reflect the population dynamics within a region of interest and drive VBD transmission models.

One example of temperature-driven disease transmission modelling (that includes explicit modelling of the vector population) was given by Piero Poletti (Bruno Kessler Foundation, Italy) for CHIKV transmission by *Ae*. *albopictus*. The model was used to investigate the basic reproduction number of a local outbreak of CHIKV in Italy in 2007, demonstrating that targeted control of the larval stages or the removal of breeding sites is more efficient at mitigating transmission than adult control in the medium-term. These types of studies are essential for informing strategies to control VBD outbreaks.

A number of other neglected tropical diseases (NTDs) have received little attention in the context of climate-driven disease modelling. Maria-Gloria Basanez (Imperial College London, United Kingdom) spoke about helminthiasis and the role of climate in disease transmission. The helminths have a variety of transmission cycles, some of which are vector-borne; these include snail-borne (e.g. schistosomiasis), blackfly-borne (e.g. onchocerciasis), and mosquito-borne (e.g. lymphatic filariasis). Directly-transmitted helminths (e.g. hookworm) will be affected by environmental conditions such as temperature, flooding, and drought. One example is that of the hookworm’s heat tolerance, which out-performs other helminths at higher temperatures, suggesting that as temperatures increase, a shift in worm population composition could occur. Other important factors for directly-transmitted helminths include changes in water storage behaviour and factors affecting host contact with water (since a number of helminths shed infective stages into water). Vector-borne helminths may be indirectly affected through changes in vector populations due to climatic conditions (such as the effects of climate on blackfly populations and onchocerciasis already discussed). It was stressed that in order to develop climate-driven models of helminthiasis, major gaps need to be addressed. These include thorough searches and syntheses of existing literature, experimental and observational data to bridge gaps identified within the existing literature, and better communication between climate modellers and impact/disease modellers. Helminth diseases of concern for the EMME include schistosomiasis (currently present in Egypt and Yemen) and onchocerciasis (current present in Yemen).

## Conclusions

Over the course of the meeting, current issues and future challenges for the field were identified by meeting participants through the presentations and discussion sessions; these key priority areas may be summarised as follows:

- *Scale*. The need to move from GCMs to RCMs, and to combine climate models with measurements on the ground, was recommended to more reliably capture meteorological conditions affecting biological systems, including those of VBD transmission dynamics.
- *Surveillance*. Better surveillance data in many countries in the EU/EMME for a variety of potential VBD vectors is essential for preparedness, response to the threat of VBD outbreaks, and planning effective vector control strategies. Although recent progress has been made in vector mapping across Europe through the VBORNET project (http://www.vbornet.eu/), many countries still do not provide information on surveillance. Nonetheless, key risk factors for VBD transmission risk in the EMME are given in Figure 2 based on meeting discussions.
- *Vector bionomics*. The lack of recent and/or local empirical vector data was identified as an important barrier to developing more accurate and reliable models of VBDs, as adaptation to local conditions needs to be reflected in model outputs. This is predominantly hampered by the current lack of funding and an apparent dearth of medical entomologists entering this field.
- *Specificity*. The need for a tailored set of environmental parameters for individual vector-disease combinations was recognised and promoted.
- *Uncertainty*. The need for transparency and more detailed uncertainty analyses, for example through ensemble approaches to disease/climate modelling, is required to improve the robustness and understanding of model output, as well as confidence in data used in planning strategies for mitigating VBDs across the EMME. This includes better communication between climate and impact modellers, an area that has improved considerably, but where further progress is undoubtedly required.

It is hoped that this meeting played a valuable role in helping to bring together experts on climate and VBDs to help develop new collaborations, identify key priorities within a wider research agenda, and, ultimately, to contribute towards debate in this sometimes controversial, but nonetheless vitally important, research field.

## Competing interests

The authors declare that they have no competing interests.

## Authors’ contributions

JW, PEP, JL, and GKC compiled this report from presentations and discussion sessions that took place during the meeting. All authors approved the final draft of this report.

